# Persistent cloaca and caudal duplication in a monovular twin, a rare case report

**DOI:** 10.1016/j.ijscr.2019.06.013

**Published:** 2019-06-12

**Authors:** Naomi Cohen, Mohamed Nagy Ahmed, Rachelle Goldfischer, Nahla Zaghloul

**Affiliations:** a Department of Pediatrics, Division of Neonatology, Cohen’s Children’s Medical Center, Northwell Health, 269-01 76th Avenue, New Hyde Park, NY 11040, USA; b Department of Radiology, Cohen’s Children’s Medical Center, Northwell Health, 269-01 76th Avenue, New Hyde Park, NY 11040, USA

**Keywords:** Cloaca, Caudal duplication, Vestigial appendage, Hydrocolpos, Duplicated genitalia, Anal atresia

## Abstract

**Introduction:**

A cloaca occurs when genitourinary tract and bowel converge into a common channel. We report a case of partial caudal duplication, persistent cloaca and vestigial appendage in a monovular female twin infant.

**Presentation of case:**

This is a monochorinonic-diamniotic twin born at 36 weeks with apgars of 9/9. She had a duplicated labia with two clitorises, and a partially formed accessory foot with 2 toes protruding from the right gluteal region. There was anal atresia and a punctate urethral opening in the right genitalia through which she voided spontaneously. X-ray of the accessory foot had rudimentary metatarsals and phalanges. There was left hydroureteronephrosis and a hydrocolpos causing severe mass effect.

On the first day of life, she had exploratory laparotomy with a diverting colostomy and mucus fistula and drainage of hydrocolpos. At 6 months of age, she had removal of the accessory foot with flap closure of the perineal defect and vesicostomy. At 15 months of age she had laparotomy for repair of cloaca, excision of presacral pelvic mass and the duplicated vulva.

**Discussion:**

Theories of etiology include failure of regression of Kovalevsky’s canal (a communication that connects the amniotic and yolk sac), an incomplete form of twinning through iatrogenic damage to the zona pellucida or a failed triplet formation from a single embryo.

**Conclusion:**

Caudal duplication with persistent cloaca and vestigial appendage is a rare and complex malformation. Having a unified surgical and medical team to preserve quality of life and to treat complications is of key importance.

## Highlights

• Persistent cloaca with caudal duplication and a vestigial appendage is very rare.
• Initial management include decompressive colostomy and drainage of the hydrocolpos.
• Staged surgery include repair of cloaca and removal of accessory foot.
• Preserving quality of life and managing complications is of key importance.

## 1 Introduction

A cloaca is a rare phenomenon in which the genitourinary tract and bowel do not separate fully and converge into a common channel. Caudal Duplication is a deformity that occurs when there is duplication of the caudal structures and notochord of the embryo. Both are rare occurrences with the incidence of cloacal malformation reported around 1:20,000–1:50,000 females [1,2] and caudal duplication as less than 1:100,000 [3]. Arnone et al have recently described a case in which both anomalies and an accessory appendage were found in a female infant [4]. We report a case of partial caudal duplication, a persistent cloaca and vestigial appendage in a monovular female twin infant. This work has been reported in line with the SCARE criteria [5].

## 2 Presentation of case

This is a case regarding a monochorinonic-diamniotic twin born at 36 weeks weighing 2130 gm. Mother was a 38 year old G3P1011. Obstetric history was significant for an ectopic pregnancy 6 years earlier and a healthy term pregnancy 2 years prior to this infant’s birth. The pregnancy was conceived through IVF with single embryo implantation. Embryo was preserved when the mother was 36 years. This Pregnancy was complicated by GDM – diet controlled, PCOS, and 2 punctate hepatic masses. Mother denies alcohol/smoking with pregnancy. Prenatal US of the fetus showed a possible left hemorrhagic ovarian cyst and possible cavum vergae.

Birth history was significant for premature rupture of membranes, Category 1 tracing, indication for cesarean section was transverse lie of the non-affected twin, female infant B

**Fig. 1:**
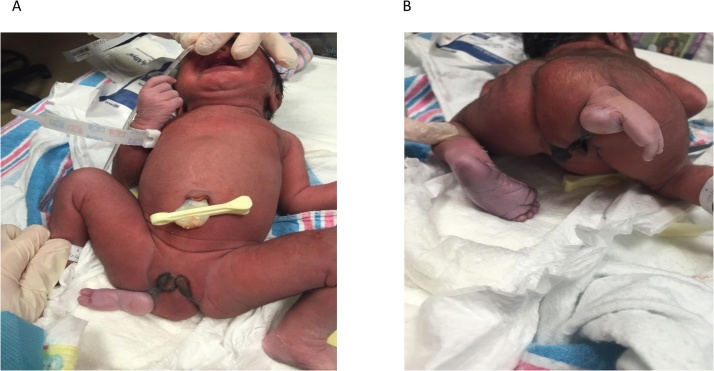
**Anterior and posterior views of infant perineum and vestigial appendage**. A: anterior view of infant perineum showing duplicated external genitalia with two clitorises, a punctate opening in the right genitalia representing urethral opening and a partially formed accessory foot with 2 toes protruding from the right gluteal region. B: posterior view of the infant perineum showing the origin of the partially formed accessory foot with 2 toes from the right gluteal region deviating the gluteal cleft to the left along with anal atresia.

The infant was born vigorous with apgars of 9/9. Her physical exam was notable for duplicated external genitalia with two clitorises, and a partially formed accessory foot with 2 toes protruding from the right gluteal region with intact reflexes (Fig. 1). There was anal atresia and a punctate opening in the right genitalia. She voided spontaneously immediately after birth.

**Fig. 2:**
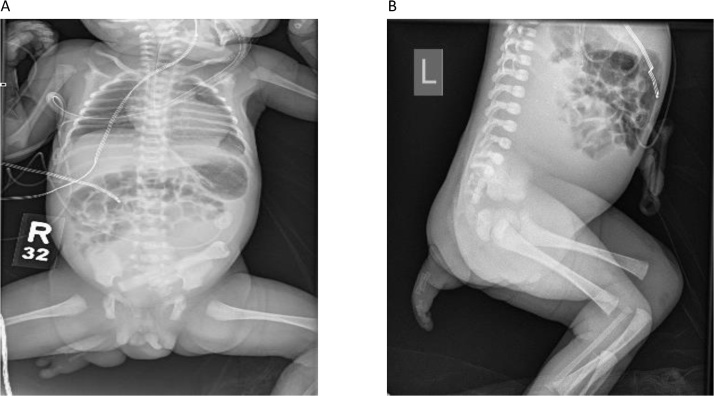
**AP and lateral X-ray views of the infant chest and abdomen**. A: AP X-ray view of infant’s chest and abdomen showing accessory foot from the right gluteal region having rudimentary 2 metatarsals and phalanges (2 toes), duplication of labia and a fused sacral vertebral body. B: lateral X-ray view of infant’s chest and abdomen showing accessory foot from the right gluteal region having rudimentary 2 metatarsals and phalanges (2 toes) and fused sacral vertebral body.

X-ray showed accessory foot had rudimentary metatarsals and phalanges, a fused sacral vertebral body and duplication of labia (Fig. 2).

**Fig. 3:**
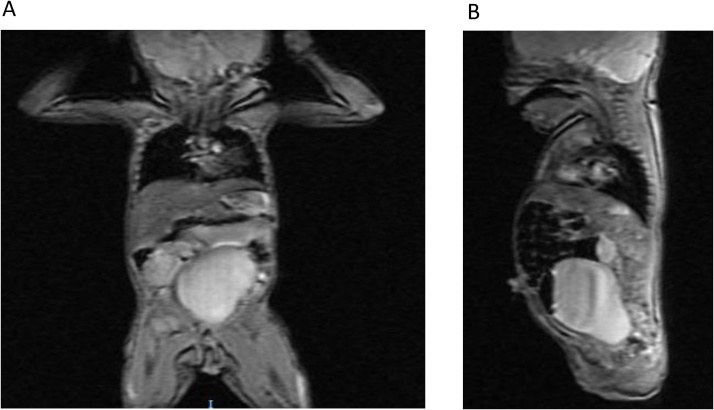
**AP and lateral MRI views of infant showing massive hydrocolpos**. A: AP MRI view of the infant showing hydrocolpos measuring 4.6 × 5.0 × 5.7 cm and causing severe mass effect on abdominal structures. B: lateral MRI view of the infant showing hydrocolpos causing severe mass effect on abdominal structures.

Renal Ultrasound showed moderate hydroureteronephrosis, the bladder was visualized, but was compressed by a large circumscribed anterior pelvic with fluid levels- likely a hydrocolpos which was confirmed by MRI measuring 4.6 × 5.0 × 5.7 cm and causing severe mass effect on abdominal structures (Fig. 3).

MRI of the spine showed that the conus medullaris terminated at L3. There was a fatty filum of the spinal cord. The spinal canal extended into the left posterior subcutaneous tissue. The sacrum was dysmorphic with the left hemisacrum coursing towards the left extending to posterior left subcutaneous tissue. The accessory appendage measured 5.6 cm in cranial caudal dimension × 4.7 cm in transverse and 1.2 cm in AP.

Other initial workup included echocardiography which showed a PDA that eventually closed within an expected period of time, an ASD which spontaneously closed prior to 2 years of age. Head Ultrasound was normal. Genetics studies were sent revealing a normal 46XX female. Microarray showed no abnormality

On DOL1 the patient went to the OR for exploratory laparotomy with creation of diverting colostomy and mucus fistula, cystoscopy, hysterostomy, and placement of a foley catheter (which was removed prior to discharge when patient was noted to be able to spontaneously void). At that time they saw the uterus was filled with mucoid fluid which was drained thereby relieving the compression on nearby structures.

At 4 months of age she presented with hydroureter and hydrometrocolpos and signs of systemic infection. She was taken to the OR for drainage of the hydrometrocolpos. Cystoscopy, vaginoscopy/hysteroscopy were performed with placement of foley catheter in the uterus and in the bladder through the cloaca. 180 ml of milky thin mucous was aspirated. Exploratory laparotomy was performed the next day to drain of peritoneal abscess as definitive tx for her peritonitis likely secondary to perforation of hydrometrocolops.

At 6 months of age the patient was taken to the OR for removal of the accessory foot with flap closure of the perineal defect. She also had a creation of vesicostomy and takedown of previously placed hysterostomy tube. Recovery was complicated by pyocolpos that was successfully treated with surgical drainage.

At 15 months of age the cloaca was repaired. Surgery consisted of cystovaginoscopy, posterior sagittal anorectal vaginal urethroplasty with laparotomy for repair of complex cloaca as well as excision of presacral pelvic mass and revision of vesicostomy and excision of duplicated vulva. Recovery was complicated by posterior sagittal wound dehiscence requiring further surgical intervention.

At 19 months of age she underwent cord detethering and excision of lipoma. Then at 23 months of age she had a revision vaginoplasty with interposition intestinal graft and revision perineoplasty and anoplasty as the neoanus was considered to be gaping and prolapsed.

Patient is currently 25 months of age with an ostomy and a vesicostomy in place. She continues to be unable to void effectively with significant reflux. She has developmental delays and has been referred to early intervention.

## 3 Discussion

Caudal duplication can encompass a variety of phenotypes. In cases of caudal duplication, leg or leg bones may be duplicated or triplicated [6,7]. Duplication of the cloaca may also be seen [6].

Dominguez et al explained that the embryologic basis for this was a developmental defect occurring to the caudal cell mass or eminence. The caudal cell mass is made of undifferentiated mesenchyme formed when the notochord and neural tube unite at 23–25 days gestation [8,9] This mass gives rise to the entire terminal spinal cord, hindgut, sacrum and pelvic soft tissues [6]. It was postulated that Kovalevsky’s canal, a communication that connects the amniotic and yolk sac fails to regress which can form fibrous bands that can divide the notochord and adjacent mesoderm or form dorsal enteric fistulous connections [7].

Other theories as include incomplete twinning. It is hypothesized that monozygotic twins occur when two primitive streaks form from one embryonic disc. It was further contemplated that the ability of a single embryo in the gastrulation stage to provide all the cells needed to form an embryo might be limited. When there are 2 organizing centers the productivity of 1 or 2 centers may be decreased. Since the caudal eminence arises late in gastrulation these may be at particular risk [6,10].

In general embryos conceived with in vitro fertilization have a higher rate of twinning. One such explanation is the iatrogenic damage to the zona pellucida allowing a blastomere to separate and potentially form a separate embryo with placental tissue [6]. It is possible that our patient represented an incomplete form of twinning after twinning had already occurred through iatrogenic damage to the zona pellucida or that this was secondary to failed triplet formation from a single embryo.

Regardless of the etiology the most important part of the patient’s care was a unified team in decision making with surgical repair being key to preserve quality of life [10]. Risk of renal failure and kidney replacement are major concerns [11,12]. Neurologic function below the level of the malformation is variable with nearly 1/3 of patients with a cloaca having tethering of the spinal cord as in our case [1,10]. Cord release will prevent neurologic dysfunction with growth, but cannot alter already established dysfunction.

Review of 141 cases revealed that prognosis for patients with cloaca show nearly 60 percent have spontaneous bowel movements and void spontaneously [1]. With a better prognosis if the common chamber is less than 3 cm [11]. Initial management as in our case focused first on decompressive colostomy and drainage of the hydrocolpos, which can be evident in up to 30% of cloacal malformations [11].

The vesitigal appendage is also an even rarer occurrence as only a handful of well-documented cases could be found in the literature [4,7].

Approximately 25% who have a cloaca will require vaginal replacement as in our patient. Typically this is done with bowel [11].

Our patient’s long-term prognosis is still unclear as she is still in the midst of her multi-surgical repair. The last VCUG did show urinary retention and reflux. Vesicostomy and ostomy are still functional. When ostomy takedown occurs patient will need daily enemas to keep her bowel clean and empty and clean urine catheterization will likely be necessary as well if she cannot void on her own.

## 4 Conclusion

Caudal duplication syndrome with a persistent cloaca and vestigial appendage is a rare and complex malformation that presents multiple questions and challenges for the medical and surgical team. Management should be directed at improving quality of life for the patient.

## Conflicts of interest

The authors (NC, MNA, RG, NZ) declare no conflict of interests or disclosures.

## Sources of funding

This research did not receive any specific grant from funding agencies in the public, commercial, or not-for-profit sectors.

## Ethical approval

Institutional ethics committee approval was taken for the publication.

## Consent

Consent for publication and photography was obtained and signed by both parents.

## Author’s contribution

NC contributed to the conceptualization, data collection and drafting of the manuscript. MNA contributed to the design and critically revised the manuscript. RG contributed to the imaging analysis and manuscript revision. NZ conceptualized, supervised and critically revised the manuscript. All authors have read and approved the manuscript for publication.

## Registration of research studies

Not applicable.

## Guarantor

Nahla Zaghloul.

## Provenance and peer review

Not commissioned, externally peer-reviewed.
